# Early Intervention Services During the COVID-19 Pandemic in Spain: Toward a Model of Family-Centered Practices

**DOI:** 10.3389/fpsyg.2021.738463

**Published:** 2021-11-11

**Authors:** Rosa Vilaseca, Fina Ferrer, Magda Rivero, Rosa M. Bersabé

**Affiliations:** ^1^Department of Cognition, Development and Educational Psychology, University of Barcelona, Barcelona, Spain; ^2^Municipal Institute of Social Services of Barcelona, Barcelona, Spain; ^3^Department of Psychobiology and Methodology of Behavioral Sciences, University of Malaga, Málaga, Spain

**Keywords:** early intervention services, COVID-19, pandemic, family-centered practices, families, professionals, telematic intervention

## Abstract

Early intervention services (EIS) worked hard to continue serving children and their families during the COVID-19 lockdown, using online applications. This study aimed to determine families’ and professionals’ perceptions of the functioning of the early intervention (EI) model in Spain during the pandemic. The study sample comprised two subsamples: 81 families of children attended at an EIS (72 mothers and 9 fathers) and 213 professionals recruited from EIS. The survey was conducted online several weeks after the end of the strict lockdown in Spain. Descriptive statistics of the questionnaire answered by families and professionals were compiled, comparisons were made between the families’ and the professionals’ responses, and the relationships with several sociodemographic variables were analyzed. The results indicated that parents who cared for their children and were fully responsible for housework, parents who had used telematic tools before the lockdown, and younger professionals had a more positive perception of the EI model and the incorporation of family-centered practices (FCP) during the pandemic. The results also showed statistically significant differences in some items between parents and professionals: for example, professionals perceived more advantages than families during the lockdown, quoting the greater participation of families in the intervention and a greater focus on families’ needs. The data obtained from professionals suggested a more positive attitude toward FCP: however, the results show that they continued to adopt a directive role in the intervention, a position that is at odds with the tenets of FCP. There is a clear need for more training if a paradigm shift to FCP is to be achieved. Families’ and caregivers’ perceptions of telerehabilitation, and their adherence to telerehabilitation programs, are discussed. The implications of this study with regard to guiding future telematic interventions and family support are also considered.

## Introduction

Over fifty years have passed since the introduction of the first early intervention services (EIS) in Spain. Today there is a wide network of universal, public, and free-of-charge EIS throughout the country, organized by the autonomous communities and run by interdisciplinary teams of professionals in the fields of health, psychology, education, speech therapy, physiotherapy, and social work. According to data provided by the *Grupo de Atención Temprana* (GAT; Early Intervention Group), there are currently over 700 EIS in Spain employing more than 4,500 professionals (Grupo de Atención Temprana, 2018).

Internationally, early intervention (EI) has come a long way in recent years, focusing less on biology and more on environmental questions, and evolving toward the integration of the biological, the psychological and the social. This is manifested mainly in the interaction of individuals with their contexts, and all these factors must be included in in each therapeutic action. In Spain, EIS have become consolidated over the years, but have also undergone major changes (some of them driven by the evolution in approaches to disability, which are no longer deficit-based but prioritize rehabilitation) and have shifted from a child-centered to a family-centered focus (Espe-Sherwindt, 2008). The scientific evidence emerging from the systemic, ecological, and transactional development model (Bronfenbrenner, 1979, 1987; Sameroff, 1983) to explain child development has also influenced their activity.

The development of EI in Spain has reflected this change in focus (García-Sánchez et al., 2014; Escorcia-Mora et al., 2016; Domeniconi and Gràcia, 2018; García-Grau et al., 2019; Gràcia et al., 2019), in response to the need for improvements in interventions (García-Sánchez, 2002; Vilaseca et al., 2004; Giné et al., 2006; Gutíez, 2010; García-Sánchez et al., 2014; Vilaseca et al., 2019a) laid down in the *Libro Blanco de la Atención Temprana* (White Book on Early Intervention; Grupo de Atención Temprana, 2005a). The *Libro Blanco* (Grupo de Atención Temprana, 2000, 2005a) and the manual of technical recommendations (Grupo de Atención Temprana, 2005b) highlight the importance of the family in the intervention. The book defines EI as “a set of interventions for children aged 0–6 years, the family, and the environment, which aim to respond as promptly as possible to the temporary or permanent needs of children with developmental disorders or those who are at risk. These interventions must be holistic and must be planned by an interdisciplinary or transdisciplinary team of professionals” (Grupo de Atención Temprana, 2000, p. 12).

Clearly, this definition highlights the need to intervene with the child, with the family, and with the community. However, theoretical models differ widely on the question of how to work with families, including those with a child with disabilities or at risk (Dalmau et al., 2017; Mas et al., 2018; García-Grau et al., 2020). Furthermore, despite the fact that international organizations such as the World Health Organization (2012), the United Nations Educational, Scientific and Cultural Organization (UNESCO, 2015), the Division for Early Childhood of the Council for Exceptional Children (Division for Early Childhood, 2014) and specialized professional associations such as the European Association on Early Childhood Intervention and the International Society on Early Intervention have all called for the incorporation of evidence-based and family-centered practices in EI, not all EIS in Spain apply a family-centered model.

In fact, most EIS in Spain still apply a child-centered approach, in which professionals intervene with the child outside his/her natural context (Dalmau et al., 2017; Vilaseca et al., 2020). According to Vilaseca et al. (2004), EI professionals spend 1% to 5% of their time working with families and 70% to 80% of their time working with the child. A more recent study (Grupo de Atención Temprana, 2011) reported a slight change in the proportions, with 14.7% of their time being devoted to families and 75% to children. Although the time spent working with families has increased, it nevertheless falls short of the international recommendations. According to Dunst and Trivette (1987, 1996, 2009), Leal (1999), Bruder (2000), Dunst et al. (2008), Espe-Sherwindt (2008) and McWilliam (2010a, b, 2011), family-centered practices (FCP) should (a) adopt an ecological and systemic approach, (b) stress the importance of the families’ natural context to promote family choices and control over desired resources, (c) empower families by placing the emphasis on their strengths, and d) develop a collaborative relationship with families – in stark contrast to the expert model, in which professionals decide how to proceed with families and what objectives to establish in their intervention programs (Serrano et al., 2017). This proposal for a change of perspective has met resistance from both families and professionals (Gràcia et al., 2019; Vilaseca et al., 2019a). For professionals, it implies a change in the way they interact with families and a shift to a model that many international authors have called family capacity-building practices (Dunst et al., 2019) – described in the early childhood intervention literature as enabling practices (Summers and Jenkins, 2001), participatory practices (Dunst and Espe-Sherwindt, 2016), engaging practices (Buckingham et al., 2016), collaborative practices (Espe-Sherwindt, 2008) and empowering practices (Dunst et al., 1988. In short, this type of intervention includes and emphasizes the support that professionals provide to parents of young children and other caregivers to promote the child’s learning and development in a model of equality and collaboration, inside contexts of everyday activities and settings (Kemp and Turnbull, 2014; Dunst et al., 2019; García-Grau et al., 2019; Vilaseca et al., 2019a, 2020). For families, this change represents a challenge as they now play an active role in decision-making rather than a passive one, and now see professionals as their partners (Turnbull et al., 2011). A literature search carried out prior to this study highlighted the barriers that EI professionals face when they shift practices from one model to another (Friedman et al., 2012; Gràcia et al., 2019; Vilaseca et al., 2019a). Coaching with parents can be challenging for professionals, as the process can easily revert to the expert-based model (García-Grau et al., 2019).

On 11 March 2020, the World Health Organization (WHO) officially declared the COVID-19 pandemic. In Spain, as in other countries, the emergency radically changed the care given to families with children attended at EIS. During the lockdown, many EIS had to suspend visits and care for families, and this undoubtedly increased the burden on parents of children with disabilities or children at risk. Behavioral regulation problems and levels of parental stress rose notably (Montirosso et al., 2021). In this new context, families had to provide 24-h care, without face-to-face access to the EIS. The most frequent concern of parents of children with disabilities was the lack of rehabilitation during the lockdown (Cacioppo et al., 2021). It is clear that social distancing seriously affects rehabilitation interventions, especially when children require physical interaction with their therapists (Provenzi et al., 2021). In Spain, in an attempt to ensure continuity of care, many of the EIS worked online, connecting with families through Skype, Zoom and other online platforms and were thus able to enter the families’ natural contexts, even if only virtually. In this way, the situation of COVID-19 provided early intervention professionals with an opportunity to implement telerehabilitation strategies inside families’ everyday contexts.

Telehealth technology has been reported to be a very useful tool in these situations, especially for rehabilitation purposes (Camden et al., 2020; Fazzi and Galli, 2020). Telehealth includes telerehabilitation, telecare, and teleconsultation. Previous research has shown that remote consultations can help to maintain closeness with therapists and help parents address concerns about their children’s development or their own psychological distress (Vismara et al., 2018). Studies of computer-mediated interventions have also shown good results with parents of children with developmental disorders or with neurodevelopmental disabilities (Kennedy et al., 2017; Balldin et al., 2018; Provenzi et al., 2020). The use of telehealth technology also facilitates access to care for families who live far away, allows the participation of the whole family by making service hours more flexible, and saves them time and money by removing the need for travel. Some assessments of teleintervention have reported fewer cancelations and greater commitment from primary caregivers (Behl et al., 2017). Indeed many studies report high levels of satisfaction among families, thus supporting the hypothesis that remote rehabilitation services can be a good alternative to direct face-to-face in-center care (Beani et al., 2020; Roggman et al., 2020; Traube et al., 2020).

During the pandemic, several tele-rehabilitation projects were set in motion in different countries for families with a child with neurodevelopmental disorders. Nevertheless, as Shorey et al. (2021) report in a review, only two studies systematically evaluated families’ responses to teleintervention during the pandemic. The first was conducted as part of the Engaging with Families in Online Rehabilitation of Children during the Epidemic (EnFORCE) telehealth program in Italy (Provenzi et al., 2021), and the second at the COVID-19 Neurodevelopmental Disorders Clinic (Summers et al., 2021) in Canada. The Italian program provided families with uninterrupted care and rehabilitation during the COVID-19 lockdown, and comprised parental support and child rehabilitation sessions conducted by the same therapists and psychologists as before the confinement, in a situation very similar to that of EIS professionals in Spain. The results of that study were spectacular: more than 80% of the parents perceived an improvement in the development of their children and, in addition, 40% reported that this type of telematic intervention had been more effective than face-to-face practice at the centers. The Canadian study (Summers et al., 2021) was a home-based consultation program implemented by a multidisciplinary team. Virtual assessments focused on problematic behaviors and lasted 60–90 min. Both these teleintervention programs were well received by most of the families. The problems identified were related to the poor quality of the internet connection, the lack of familiarity with telematic tools, interruptions, and difficulties following the instructions (Shorey et al., 2021).

In Spain, for researchers into early intervention practices, the COVID-19 pandemic brought an opportunity to move toward a family-centered model, despite all the difficulties and challenges that the situation posed. Some professionals in early intervention already had training and experience in teleintervention before the pandemic, while for others it was an entirely new experience. We should highlight certain support initiatives, such as the guide prepared by Plena Inclusión (2020). This situation made us reflect on whether, despite the terrible consequences of the COVID-19 pandemic, it might have given professionals and families the impetus to adopt the principles of family-centered practices – a change of perspective which had been proposed for so long in Spain (Tamarit, 2015; Dalmau et al., 2017; Mas et al., 2018; Gràcia et al., 2019; Vilaseca et al., 2019a).

Therefore, the general aim of this study was to identify the changes in the intervention methodology used with families receiving EIS in Spain in the new scenario created by the COVID-19 pandemic. More specific aims were: (a) to analyze the families’ and professionals’ perceptions of the intervention model received and implemented during the pandemic lockdown; (b) to explore the relation of certain sociodemographic variables and the families’ and professionals’ perceptions of the intervention model; (c) to identify any differences between families’ and professionals’ perceptions of the intervention model in this exceptional context.

## Materials and Methods

### Participants

Convenience sampling – a type of non-probabilistic sampling- was used to select participants who were recruited from several EIS in Spain. Two inclusion criteria were applied: families had to have a child attended by an EIS at the time of receiving the survey, and professionals had to have been working in the EIS for at least one year prior to the lockdown. The participants were volunteers who met the inclusion criteria and responded to a request to take part (see section “Procedure”).

The study sample comprised two subsamples: 81 families and 213 professionals (see Tables 1, 2). The subsample of families (parents) was composed of 72 mothers (88.9%) and 9 fathers (11.1%). The mothers had a mean age of 38.1 years (*SD* = *6.9*) and the fathers of 39.9 years (*SD* = *14.9*). Most parents were married or living with a partner (90.1%). Half of them had completed high school (46.9%) or had a university degree (39.5%). They were either employed full-time (42%), employed part-time (22.2%) or cared for their children and were fully responsible for housework (19.8%). The majority were from Catalonia (79%), 18.5% were from Castilla La Mancha and 2.5% from Andalusia.

**TABLE 1:** Demographic characteristics of the family members who answered the survey and the child attended at an EIS (*n* = 81).

Characteristics of the person who answers	*n* (%)	Characteristics of child attended at an EIS	*n* (%)	Characteristics of attention in EIS before COVID-19	*n* (%)
Sex		Sex		Frequency of sessions	
Female	72 (88.9)	Female	19 (23.5)	Twice-weekly	4 (4.9)
Male	9 (11.1)	Male	62 (76.5)	Weekly	30 (37.0)
Age (years)		Age (months)		Every two weeks	20 (24.7)
< 29	6 (6.2)	< 24	17 (21.7)	Monthly	15 (18.5)
30–39	37 (45.4)	25–48	34 (43.1)	Others	3 (3.6)
40–49	36 (43.1)	49–59	18 (22.9)	They had not started	9 (11.1)
> 50	4 (4.8)	> 60	11 (14.1)	Session length (minutes)	
*Employment status*		Degree of disability		60	44 (54.2)
Employed full-time	34 (42.0)	Mild	52 (64.2)	45–50	28 (34.6)
Employed part-time	18 (22.2)	Moderate	21 (25.9)	< 40	9 (11.1)
Homemaker	16 (19.8)	Severe	8 (9.9)	Session mode	
Others	13 (16.0)	Therapy received at the EIS		Professional and child	40 (49.4)
Level of education completed		Speech therapy	33 (41.3)	Professional talks with the family at the end of the session	11 (13.6)
Primary or no studies	11 (13.6)	Psychology	24 (30.0)	Professional involves the family	19 (23.5)
Secondary	38 (46.9)	Physiotherapy	16 (20.0)	Professional goes to child’s home	1 (1.2)
University degree	32 (39.5)	Others	7 (8.8)	Others	10 (12.3)

**TABLE 2:** Demographic characteristics of professionals (*n* = 213).

Characteristics	n (%)	Characteristics	n (%)
Sex		Discipline	
Female	204 (95.8)	Physiotherapy	58 (27.2)
Male	9 (4.2)	Psychology	75 (35.1)
Age (years)		Speech therapy	45 (21.2)
< 29	23 (10.7)	Psychomotor skills	13 (6.2)
30–39	74 (34.8)	Social work	6 (2.8)
40–49	69 (32.4)	Pedagogy/psychopedagogy	6 (2.8)
50–59	29 (13.5)	Others (neuropediatrics, occupational therapy…)	10 (4.7)
> 60	16 (7.5)	Number of people in team	
Missing	2 (1.0)	< 5	37 (17.3)
Years of experience		6–10	55 (25.9)
< 2	30 (14.1)	11–15	42 (19.8)
2 - 5	39 (18.3)	16–20	33 (15.4)
6 - 10	25 (11.7)	21–25	34 (16.0)
> 10	119 (55.9)	>26	12 (5.6)

Among the children, 76.5% were male and 23.5% were female, with an age range from 7 to 68 months (*M = 40.5, SD = 16.4*). The degree of intellectual disability (ID) was mild (33 – 64%) in 64.2%, moderate (−65 – 74%) in 25.9% and severe (> 75%) in 9.9%. In Spain, assessment of the percentage of disability is a standardized process carried out by a government agency, the Valuation and Guidance Services for People with Disabilities. ID is graded as mild, moderate or severe. A total of 41.3% of children received speech therapy, 30% psychological support and 20% physiotherapy. More than half (54.2%) received 60-min sessions at the EIS, either once a week (37%) or every other week (24.7%) before the pandemic. Regarding the format of the pre-pandemic sessions, almost half of the family subsample stated that the professional attended exclusively to the child (49.4%), 23.5% stated that s/he involved the family and only 1.2% reported that s/he came to their home. Before the onset of the pandemic, it tended to be the mother who took the child to the EIS (49.4% alone or 29.6% together with the father). Most families (64.2%) had no online contact before COVID-19.

The subsample of professionals comprised 204 women (95.8%) and 9 men (4.2%), with a mean age of 41 years (*SD = 10.9*). With regard to their specializations, 27.2% were physiotherapists, 35.1% psychologists and 21.2% speech therapists. Most had over five years of experience working at an EIS (67.6%). Most (63%) worked in a team with a maximum of 15 members. Most of the participating EIS were based in Catalonia (58.7%), with 23% in Castilla La Mancha, 15% in Andalusia, and 11% in Valencia.

### Instruments

Once the family or professional received the document via e-mail and clicked on the link, they were given information about the nature and purpose of the survey on the first page. Subsequently, if they agreed to participate, they were taken to the sociodemographic questionnaire on the following page. The second part of the survey asked about their perceptions of how the intervention methodology at the EIS had changed as a result of COVID-19.

The family version of the *Brief sociodemographic questionnaire* compiled data on marital status, educational attainment, employment status, number of people living in the home, gender and age of their child, degree of disability, and frequency of attention in EIS before lockdown. The version for professionals compiled data on their field, number of EIS professionals at their center, years of work experience, and so on.

The *Questionnaire on EIS interventions in times of COVID-19 for families (Intervención en los CDIATs en tiempos de COVID-19 para familias)* was developed *ad hoc* for this study. The main objective was to evaluate families’ perceptions of the changes in the way professionals intervened with their children since the pandemic.

The *Questionnaire on EIS interventions in times of COVID-19 for professionals (Intervención en los CDIATs en tiempos de COVID-19 para profesionales)* also developed *ad hoc* for this study, assessed professionals’ perceptions of changes in the methodology of intervention with families and children since the pandemic.

Both surveys were translated into Catalan for people from Catalonia and Valencia. Initially, the measures consisted of 14 items. In the version for families, the items measured aspects related to the use of telematic means (video calls, videos, e-mails, etc.) with the EIS professional as a result of the lockdown. For example, some questions explored whether interventions carried out through a video call allowed family members to talk in more detail about daily routines or about the child’s functioning at home (item 1) or participate more in the intervention (item 2), or whether the professional continued to decide what to work on with the child at home (item 3).

In the version for professionals, these items measured, for example, whether the use of telematic means allowed them to learn more about the child’s natural context (item 1) and to focus on the needs of the entire family and not just the child (item 5), or asked about the need for further training to intervene in the natural context (item 14). Table 3 (families) and Table 4 (professionals) display all the items for both instruments.

**TABLE 3:** Exploratory factor analysis and descriptive statistics for the questionnaire answered by the families (*n* = 81).

	Factor loading	*M*	*SD*	*t*_(80)_
1. Connecting by video call with the EIS professional has allowed us to talk more than before about our daily routines, the child’s functioning at home, our daily organization, etc.	0.566	2.70	0.99	1.84
2. During the follow-up via video call, we were able to participate more and contribute our opinions on what to work on with our child, and on the difficulties encountered on a day-to-day basis, etc.	0.737	2.69	0.97	1.77
3. The EIS professional has continued to propose and decide which aspects we can work on or strengthen with our child at home.	0.853	3.24	0.88	7.57***
4. The EIS professional has guided us to find new ways to use the material we have at home.	0.864	3.17	0.93	6.49***
5. We feel that the EIS professional has taken our emotional needs as a family more into account during lockdown than before lockdown.	0.581	2.61	1.01	1.03
6. In addition to caring for the child, we have been able to discuss other situations that affect us as a family (e.g., symptoms of anxiety or depression as a result of COVID-19, concerns about the current economic and employment situation, etc.).	0.727	2.91	1.07	3.46**
7. In the video call sessions, all members of the family (mother, father and/or siblings) have participated, whereas before we were not able to do this.	0.569	2.37	1.05	–1.10
8. The EIS professional has given us guidelines on what we can do as parents to promote our child’s development at home.	0.861	3.22	0.93	6.94***
9. The sessions lasted as long as before lockdown.	0.652	2.98	0.99	4.41***
10. Unlike before, the professional has added our opinions to the work plan with our child.	0.677	2.54	1.00	0.39
11. The online sessions have continued to be led by the professional.	0.732	3.08	0.96	5.47***
12. We are satisfied with the care we have received from EIS during lockdown.	0.839	3.23	0.95	6.94***
Total score	0.566	2.90	0.91	5.09***

**p < 0.01 ***p < 0.001 One sample *t-*test (mean value = 2.5).

**TABLE 4:** Exploratory factor analysis and descriptive statistics for the questionnaire answered by the professionals (*n* = 213).

	Factor loading	*M*	*SD*	*t _(212)_*
1. Connecting by video call with families or through videos has allowed me to increase my knowledge about specific aspects of family dynamics in the child’s natural context, daily routines, how the child functions at home, etc.	0.755	3.15	0.76	12.34***
2. In the intervention sessions with families and children at home, families have been more participative (they have contributed their opinions on aspects to work on, difficulties encountered, etc.).	0.733	2.99	0.72	9.99***
3. I have been able to propose, with input from the parents, functional objectives concerning what the child and family do at home.	0.710	3.08	0.76	11.22***
4. I have guided families to identify new ways to use the material they already have or the routines they already do to support their child’s development.	0.754	3.26	0.69	15.95***
5. I have been able to work with the families during this period of lockdown, based on their needs.	0.761	3.33	0.71	17.09***
6. In addition to caring for the child, I have been able to think of specific goals for the caregivers (e.g., related to the presence of symptoms of anxiety or depression as a result of COVID-19, concerns regarding their current economic and employment situation, etc.).	0.655	3.00	0.79	9.35***
7. I have had the opportunity to see the entire family unit and involve all the members, since the mother, father and/or siblings were present in the sessions we held through video calls.	0.649	2.68	0.87	3.07***
8. I have been able to promote parent-child interactions that enhanced the child’s development at home.	0.716	3.03	0.71	11.00***
9. I have had the opportunity to give positive feedback to parents about their interactions with their child and enhance their strengths.	0.836	3.35	0.74	16.84***
10. If siblings were present in the video call sessions, I was able to observe the relationship between siblings.	0.637	2.76	0.94	4.08***
11. From now on, I want to continue using tools to work with families and children in the natural context.	0.714	3.14	0.81	11.43***
12. The experience of lockdown has made me rethink the way I work with families and children.	0.569	2.87	0.89	6.05***
13. The experience of lockdown has made me realize that I need more training on how to intervene with families and children in their natural context.	0.512	2.88	0.87	6.48***
Total score		3.04	0.54	14.54***

**p < 0.01 ***p < 0.001 One sample *t-*test (mean value = 2.5).

Families and professionals were asked to state how far they agreed with each of the items on a Likert scale ranging from 1 (strongly disagree) to 4 (strongly agree). The Cronbach’s alpha coefficient of the final questionnaire for families, composed of 12 items, was 0.915, and the final questionnaire for professionals, with 13 items, was 0.906, indicating acceptable internal consistency (Taber, 2018).

### Procedure

During the pandemic, the safest way to collect data was through an online survey. We conducted a nationwide cross-sectional study through an electronic survey in Google Forms (Google LLC, Mountain View, CA, United States). We prepared two surveys: one for families and the other for professionals. This study was approved by the Network of Ethics Committees in Universities and Public Research Centers in Spain in accordance with the International Ethical Guidelines for Health-related Research Involving Humans and written informed consent was obtained from parents and professionals prior to data collection.

First of all, we contacted two organizations that manage EIS in Spain and Catalonia, the Spanish Association for Early Childhood Intervention (AEIPI) and the Catalan Association of Early Intervention (ACAP). We sent them a document via e-mail with a brief explanation of the project, the objectives and methodology, and a link to a fuller explanation of the project, the informed consent form, a brief sociodemographic questionnaire and the survey. The associations sent the information to all affiliated members. Participation in the study was voluntary and anonymous, and participants did not receive any financial compensation.

Parents or professionals who agreed to participate, answered the online survey, which was available for approximately three weeks (from 11 June to 7 July 2020). The survey took approximately 15 min to answer. At the end of the survey both families and professionals had the possibility to add observations or comments and to contact the researchers if they had questions.

### Data Analysis

An exploratory factor analysis was conducted to identify the underlying dimensions of each of the two versions of the *Questionnaire on EIS interventions in times of COVID-19.* Data for each questionnaire underwent Principal Component Analysis (PCA) with Varimax rotations.

Cronbach’s alpha coefficients were computed for each scale to provide an indicator of internal consistency of the measures. For item analysis, we calculated Cronbach’s alpha if an item was deleted, and also discrimination indexes, obtained as the corrected correlation of the item score with that of the corresponding scale. Total scores were obtained by calculating the mean for the items on each scale.

Descriptive statistics (mean and standard deviation) were computed for each of the questions answered by professionals and families. Each item was scored on a four-point Likert-type scale (1: Strongly disagree, 2: Disagree, 3: Agree, 4: Strongly agree). A one sample *t-*test was used to determine whether the mean score of each item was different from 2.5 (the midpoint of the scale). In addition, differences between professionals and families were analyzed by comparing the mean scores of the items with similar content for both groups, via an independent sample *t*-test.

To study the relationship between each of the demographic variables and the total scores on the questionnaires, a bivariate analysis was conducted. For categorical demographic variables, total scores were compared via an independent sample *t*-test (to compare two means) or One-Way ANOVA (for more than two means), followed by *post hoc* pairwise comparisons. Relationships between continuous demographic variables and total scores were examined via Pearson product-moment correlation coefficients (or Spearman’s correlation coefficients for ordinal demographic variables). For the bivariate analysis, effect size was calculated by Cohen’s *d*, Eta squared (η^2^), or *R* squared.

Finally, variables whose effect was found to be statistically significant in the previous bivariate analyses were included in a linear regression model to predict total score on the questionnaires. IBM SPSS Statistics (version 26.0 for Windows) was used for all statistical analyses. Missing data were handled by pairwise deletion. For all analyses, statistical significance was defined as *p* < 0.05.

## Results

### Factor Analysis and Reliability of the Questionnaire for Families

Principal Component Analysis (PCA) was used to explore the dimensionality of the Questionnaire on EIS interventions in times of COVID-19 for families. According to the Unidimensionality Index, *UI* = (λ_1_−λ_2_)/(λ_2_−λ_3_) = 19.45, the items clearly satisfied unidimensionality (Slocum-Gori and Zumbo, 2011). All item loadings were greater than 0.50, except items 12 (“We like to use our own material rather than that of the EIS, because we can use it every day and it helps our child”) and 13 (“Our child has received less attention than before lockdown”) with loadings lower than 0.30. Also, Cronbach’s alpha coefficient increased if items 12 and 13 were deleted. For these reasons, these two items were removed from the questionnaire and items were renumbered accordingly.

The final questionnaire (comprising 12 items) underwent PCA again. The Kaiser-Meyer-Olkin measure of sampling adequacy was 0.87, and Bartlett’s test of sphericity was highly significant (*p* < 0.001), indicating that the data were suitable for the analysis. The one-factor solution accounted for 53.2% of the total variance. All item loadings were greater than 0.50 (see Table 3).

Cronbach’s alpha coefficient was computed to assess the questionnaire’s internal consistency. For item analysis, we calculated Cronbach’s alpha if an item was deleted, and homogeneity indexes, obtained as the corrected correlation of the item score with the total score. In this sample, Cronbach’s alpha was 0.915, and it decreased if any of the items were deleted. Homogeneity indices were greater than 0.50 for all items. Thus, the final questionnaire seemed to show a unidimensional structure with a high internal consistency.

### Descriptive Statistics of the Questionnaire for Families

A total score was obtained by calculating the mean score of the 12 items included in the questionnaire. Therefore, total scores (like the item scores) ranged from 1 to 4. Table 3 shows descriptive statistics (mean and standard deviation) of the total scores, and each of the items answered by families (*n* = 81). In eight items, the difference between the mean item score and the midpoint of the scale (2.5) was statistically significant (*p* < 0.01). This result indicates that, on average, families agreed with the statement made in the questions. In particular, the content of those items was related to professional guidelines promoted to foster the child’s development at home (item 8), proposing what to work on (item 3) and using the material they had available in the home (item 4). In addition, families agreed that they could discuss other situations affecting them at family level (e.g., symptoms of anxiety or depression because of COVID-19) (item 6). They also reported that the virtual sessions continued to be led by the professional (item 11) and lasted as long as they had done before lockdown (item 9). In general terms, they were satisfied with the care they received from the EIS during lockdown (item 12).

In contrast, the mean score for six of the items was not significantly different (*p* > 0.05) from the midpoint of the item scale (2.5). This means that families did not clearly agree or disagree with the content of the items. Specifically, they did not report that virtual sessions via a video call allowed them to talk more than before about their daily routines, the child’s functioning at home, etc. (item 1), or that they could participate more and contribute their opinions on aspects to work on with their child (item 2). Nor did they particularly agree that their emotional needs as a family were taken into account more than before the lockdown (item 5), that all members of the family participated whereas previously they had not been able to (item 7) or, that their opinions were now added to the work plan (item 10). Therefore, in certain aspects such as the duration of the sessions, the participation of all family members, and the involvement of professionals in other areas (as well as the emotional needs of families), families did not perceive a significant change compared with the pre-lockdown period.

In any case, the total score differed significantly (*p* < 0.001) from the midpoint (2.5), indicating that (on average) families agreed with the items on the questionnaire, since the mean total score (*M* = 2.90) was approximately equal to the third point of the Likert-type scale (3: “Agree”).

### Sociodemographic Factors and Total Score on the Questionnaire for Families

The relationship between sociodemographic factors and the total score on the family questionnaire was analyzed. Specifically, the following sociodemographic factors were included in the study: parent’s age and gender, marital status, educational level, employment status, number of people living at home, age and gender of their child, degree of intellectual disability, and frequency of visits to the EIS before lockdown. Parents were also asked whether they had online contact with the EIS before lockdown, and whether they used telematic tools.

The results showed a statistically significant effect of employment status on the total score on the family questionnaire (Welch’s *F*(2, 38.4) = 4.79; *p* < 0.014; η^2^ = 0.125). The highest mean total score for the family questionnaire was found in parents who cared for their children and were fully responsible for housework (*M* = 3.32; *SD* = 0.57), followed by those who were employed part-time (*M* = 2.97; *SD* = 0.42), and those employed full-time (*M* = 2.68; *SD* = 0.84). Pairwise comparisons (via the Games-Howell test) showed higher total scores in parents fully responsible for housework than in those employed full-time (*p* < 0.05); no differences were found between the other categories of the variable. Using Cohen’s (1988) benchmarks for interpreting effect sizes, the effect of employment status on total questionnaire score can be considered as medium (0.06 < η^2^ < 0.25).

The results also showed a relationship between the use of telematic tools prior to lockdown and the total questionnaire score for families (Welch’s *t*(11.52) = 4.22; *p* = 0.001; Cohen’s *d* = 1.54). Parents who used telematic tools prior to the pandemic had a higher mean questionnaire score (*M* = 3.04; *SD* = 0.56) than those who had not used them (*M* = 1.96; *SD* = 0.82). In accordance with Cohen (1988), the effect of the use of telematic tools on the total questionnaire score of the families can be considered as large (*d* > 0.50).

The other demographic variables (parents’ age and gender, marital status, educational level, number of people living at home, age and gender of their child, degree of intellectual disability, and frequency of visits to the EIS before lockdown) did not show significant effects (*p* > 0.05) on the total score of the questionnaire answered by the families.

Sociodemographic factors whose effect was found to be statistically significant in the previous bivariate analyses (*p* < 0.05) were included in a multiple linear regression model to predict the total score on the family questionnaire. Two potential factors were taken into account: (1) parent’s employment status, and (2) use of telematic tools prior to the pandemic. The results (Table 5) indicate that total scores on the family questionnaire could be predicted by a linear combination of the parent’s employment status and previous use of telematic tools. In particular, high total scores on the questionnaire corresponded to parents who cared for their children and were fully responsible for housework (versus those in full-time employment), and who had used telematic tools before the pandemic. The regression model accounted for 35.7% of the variance of the total questionnaire scores (adjusted *R*^2^ = 0.357).

**TABLE 5:** Linear regression model on total scores of the questionnaire for families.

Predictor	Estimate	*SE*	*t*	*p*
Intercept	0.524	0.426	1.23	0.224
**Employment_status:**				
Employed part-time – Employed full-time	0.214	0.172	1.24	0.220
Homemaker – Employed full-time	0.428	0.183	2.34	0.023
**Use of telematic tools:**				
Yes – No	1.183	0.227	−5.21	< 0.001

### Factor Analysis and Reliability of the Questionnaire for Professionals

Dimensionality of the Questionnaire on EIS interventions in times of COVID-19 for professionals was explored by PCA. According to the Unidimensionality Index, *UI* = (λ_1_−λ_2_)/(λ_2_−λ_3_) = 25.7, the items clearly satisfied unidimensionality (Slocum-Gori and Zumbo, 2011). All item loadings were greater than 0.50, except item 11 (“Before the opportunity to do this follow-up with families and children at home, it was difficult for me to see the importance of an intervention in the natural, family-centered context”) with a loading lower than 0.30. Also, Cronbach’s alpha coefficient increased if item 11 was deleted. For these reasons, this item was excluded from the questionnaire, and items were renumbered accordingly.

Principal component analysis was again conducted on the final questionnaire, which comprised 13 items. The Kaiser-Meyer-Olkin measure of sampling adequacy was 0.904, and Bartlett’s test of sphericity was highly significant (*p* < 0.001), indicating that the data were suitable for the analysis. The one-factor solution accounted for 48.6% of the total variance. All item loadings were greater than 0.50 (see Table 4).

With respect to the internal consistency of the questionnaire for professionals, Cronbach’s alpha was 0.906, and it decreased if any of the items were deleted. Homogeneity indices were greater than 0.40 for all items. Therefore, the final questionnaire showed a unidimensional structure with a high internal consistency.

### Descriptive Statistics of the Questionnaire for Professionals

A total score was obtained by calculating the mean score of the 13 items included in the questionnaire. Total scores ranged from 1 to 4. Table 4 shows descriptive statistics for the total score and each question answered by professionals working at an EIS (*n* = 213). In all the items and the total score, the mean score was statistically different (*p* < 0.001) from the midpoint of the item scale (2.5). Therefore, on average, professionals agreed with the statements contained in all the questions. This indicates that connecting by videoconference with the families and children had positive consequences for the professionals, such as being able to identify specific aspects of the family dynamics, daily routines or the functioning of the child in his/her own home, etc. (items 1 to 10). This new way of connecting with families, caused by the lockdown situation, has led EIS professionals to rethink their way of working and has encouraged them to intervene with families and children in their natural context (items 11 to 13).

### Sociodemographic Factors and Total Score on the Questionnaire for Professionals

Several sociodemographic factors were included in the study: professionals’ gender and age, years of experience working at an EIS, professional field, and number of members in the team. A statistically significant Pearson’s correlation coefficient was found between cognitive professionals’ age and total scores on the professionals’ questionnaire (*r* = −0.144; *p* = 0.036). This indicates that younger professionals showed higher scores on the questionnaire than their older peers. The other demographic variables included in this study had no statistically significant effect on the total scores on the professionals’ questionnaire.

**FIGURE 1:**
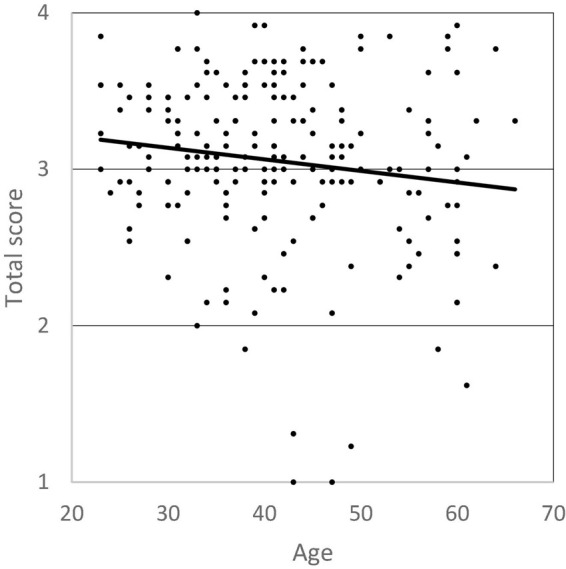
Relationship between professionals’ age and total score on the questionnaire.

Professionals’ age was included in a linear regression model to predict total scores on the professionals’ questionnaire. Results (Table 6) indicate that total scores could be predicted by professionals’ age, although the regression model accounted for only 1.6% of the variance of the total questionnaire scores (adjusted *R*^2^ = 0.016). Indeed, the regression line (represented in Figure 1) shows a slight downward trend, indicating that older professionals had lower total scores on the questionnaire, although the effect size can be considered as low.

**TABLE 6:** Linear regression model on total scores of the questionnaire for professionals.

Predictor	Estimate	*SE*	*t*	*p*
Intercept	0.524	0.426	1.23	0.224
Professionals’ age	1.183	0.227	−5.21	< 0.001

### Comparison Between Professionals and Families

Six questions on the professionals’ and families’ questionnaires were almost identical (items 1, 2, 3, 4, 6, and 8). For each of the questions, the difference in means between the two groups was analyzed. The data analysis was conducted via independent samples *t*-test. The results showed statistically significant differences for two items: the first (*t*_(118.2)_ = 3.65; *p* < 0.001) and the second (*t*_(115,4)_ = 2.56; *p* = 0.012). On these two questions, the professionals had higher average scores than the families. In fact, among the professionals (see Table 4), the mean score on these questions was significantly higher than 2.5 (*p* < 0.001), which means that most of them agreed with the statements in these items. In the case of family members (see Table 3), the mean for the same questions was not significantly higher than 2.5 (*p* > 0.05). Thus, the professionals, but not the family members, did perceive certain advantages during the lockdown: for example, they learnt about specific aspects of the family dynamics in the child’s natural context (item 1) and felt that families participated more actively and could give their opinions on aspects to be worked on, difficulties encountered, etc. (item 2).

In the four other remaining questions, no statistically significant differences were found between the groups (*p* > 0.05); that is, both professionals and family members generally agreed with the content of the issues raised. Both groups agreed that the professional was able to suggest what the child and family could work on at home (item 3), guide families to find new ways to use the material (item 4), attend to other situations affecting the family (such as symptoms of anxiety or depression as a result of COVID-19, worries about money and employment etc.) (item 6), and promote parental interactions that enhanced the child’s development in their own home (item 8).

## Discussion

The two surveys in this study recorded information on families’ and professionals’ perceptions of the EI methodology used during the COVID-19 pandemic in Spain. Our aims were to analyze and compare these perceptions and to explore the relation between them and certain sociodemographic variables.

Our general hypothesis was that videoconferencing would promote the use of family-centered practices (FCP), by bringing professionals closer to the family context. We expected both families and professionals to perceive an increased use of FCP in the intervention model, However, the results of the study did not clearly indicate this; rather, they lend support to the idea proposed both in the United States and in Europe (Bezdek et al., 2010; Tomasello et al., 2010; Serrano et al., 2017) that the family-centered approach (FCA) is not easy to apply. With respect to the influence of sociodemographic variables on these perceptions, our study was exploratory.

In relation to the families, most respondents were mothers (almost 90%). Although the participation of the father at EIS is increasingly recommended to promote children’s early development (Tamis-LeMonda et al., 2004; Cabrera et al., 2011) and although systemic and ecological theories of development emphasize the dynamic and interdependent nature of the family unit (Bronfenbrenner, 1979, 1987; Sameroff, 1983), it still tends to be mothers who organize and orchestrate the needs of children with a disability (McWilliam and Er, 2003; Rantala et al., 2009; Vilaseca et al., 2020). If the aim is to move toward new, more systemic and ecological models of intervention, the figure of the father or other caregivers such as grandparents, uncles/aunts and older siblings must be incorporated in EIS (Davys et al., 2017; Crnic et al., 2019; Vilaseca et al., 2019b).

Furthermore, the data obtained from the families suggest that in almost 50% of cases the professional is the person who works with the child. Professionals go to family homes only 1% of the time and only involve the family into their intervention model in 2% of cases. These results are consistent with other studies in Spain (Vilaseca et al., 2004; Grupo de Atención Temprana, 2011; Escorcia-Mora et al., 2018; Mas et al., 2018; García-Grau et al., 2019) but are clearly at odds with most model home visiting programs carried out in the United States, which focus on working in the family’s home (Sama-Miller et al., 2017).

Of the 12 items evaluated by the families, the difference between the mean item score and the midpoint of the scale was statistically significant in seven. Families were satisfied with the professional attention received during the lockdown (item 12), and the duration of the sessions did not change (item 9). They felt that professionals offered guidance to use the home materials in innovative ways to improve the child’s development (item 4) and they had the opportunity to talk with the professional about aspects of family life other than attending to the child, such as the emotional impact of COVID-19 or economic and employment problems (item 6). Our findings are consistent with previous studies using a telehealth family-centered rehabilitation program for children with disabilities during the COVID-19 lockdown (Provenzi et al., 2021). In that study, more than 86% of parents reported increased feelings of engagement, self-relevance, perceived support, and recognition of their role in childcare and development. Other studies carried out in families with children with neurodisabilities also reported a high level of satisfaction in relation to the use of telerehabilitation (Beani et al., 2020; Fazzi and Galli, 2020). Likewise, in studies carried out with young children with autism, cerebral palsy, and other neurodevelopmental disorders, parents also reported qualitative benefits of teleintervention, such as greater parental self-efficacy and empowerment to interact with their children in their natural context (Little et al., 2018; Wallisch et al., 2019). For us, these results support the use of telerehabilitation to implement best practices for children with disabilities in order to promote their learning and development in their habitual contexts.

Finally, and not at all surprisingly, parents showed significant agreement regarding other items that reflect an expert-focused EI model. They felt that professionals continued to play a directive role in the identification of developmental outcomes to promote at home (item 3) and that they continued to lead the virtual sessions (item 11). Parents felt that the professional provided concrete guidelines for promoting the child’s development at home (item 8), which reflects a child-centered EI model. These results indicate that families continue to lack control over the EI practices that their child receives. We agree with García-Grau et al. (2019) that the Libro Blanco (Grupo de Atención Temprana, 2005a) and the Technical Recommendations for Early Intervention in Spain (Grupo de Atención Temprana, 2005b) do not contain enough practical recommendations to aid the transition from a child-centered approach to a family-centered approach. The families’ responses suggest that the specific actions carried out by the professionals had little in common with the participatory practices recommended in family-centered services, despite the opportunities that the use of remote technology could offer them.

In other items that referred directly to videoconferencing and to relevant characteristics of FCP, parents did not express clear agreement or disagreement. They did not report that videoconferencing allowed them to speak more specifically than before about daily routines, about the functioning of the child at home or about the family’s daily organization (item 1). Videoconferencing did not increase their participation in the definition of intervention aims or daily difficulties in attaining these aims (items 2 and 10), attention to the emotional needs of the family (item 5) or the participation of all family members (item 7). These results indicate that although the introduction of remote technologies may have been useful during the pandemic for daily clinical practice and for the treatment of children with neurodisabilities, in Spain, professionals still require training in their application. The potential of telerehabilitation is increasing exponentially, both in European and further afield (Montirosso et al., 2020; Traube et al., 2020; Provenzi et al., 2021; Summers et al., 2021).

On the other hand, the results of our study indicate that total scores on the families’ questionnaire can be predicted by a linear combination of parents’ employment status and their previous use of telematic tools. Parents who answered the questionnaire (mothers in almost all cases) and who cared for their children and did the housework had a more positive perception of the intervention during the lockdown. This interesting finding can probably be attributed to the role that is assigned to mothers within families with children with developmental delays. According to Elam et al. (2017), mothers tend to assume greater responsibility in the management of family tasks, such as organizing daycare and following the indications of the EI professional. This in no way implies that mothers should be advised not to do paid work: it merely indicates that they may be more aware of the characteristics of the intervention being carried out. Today in Spain, mothers still spend more time with their children than fathers. In the case of children with disabilities, they accompany them to the EIs, the pediatrician, and school meetings (Vilaseca et al., 2020). Indeed, in most western countries, women are still the primary caregivers, especially in the case of families with a child with a disability (Bianchi et al., 2012). It is important to encourage mothers with children with developmental delays to work outside the home, since this activity can reduce emotional distress in families with children with disabilities (Vilaseca et al., 2014), and it does not in any way conflict with the FCP guidelines.

Not surprisingly, parents accustomed to using computer resources before the COVID-19 pandemic had a more positive perception of the online intervention during the lockdown. Those results are consistent with previous studies assessing factors that either promote or hamper the use of telehealth. Difficulty in accessing technical resources is one of the main reasons for rejecting teleintervention (Kraljević et al., 2020).

Most EI professionals who responded were women, almost 96%; most were aged between 30 and 49 years old and over half had more than ten years of experience working in EI. Most teams comprised six to 10 professionals from different fields.

The survey results showed that the professionals (unlike families) expressed agreement with all items, and the results were all statistically significant. It seems that the pandemic and the use of video calls or videoconferences brought them closer to the families and helped them to understand their needs and adjust to them (items 1, 5 and 6). Likewise, they felt that families were more participative than before (item 2); they were able to propose functional objectives adapted to families’ routines involving all family members, including siblings (items 3, 7, and 10) and could guide them to identify new uses for the materials they already had (item 4). Professionals based their practice on parenting, promoting positive interactions between mothers, fathers, and children to promote child development (item 8), and on giving feedback to enhance family strengths (item 9). These results conflict with the findings of a study of 250 EI professionals carried out in the pre-COVID era by García-Grau et al. (2019), who found the most difficult practices carried out with families to be the identification of family support, addressing families’ needs with routines, and scheduling family visits in a way that adapted to the needs of all members. Studies continue to show that there is a huge difference between family-centered best practices and what professionals do on a day-to-day basis (Espe-Sherwindt and Serrano, 2016). Although it seems that the pandemic has made EI professionals in Spain pay more attention to the principles of FCA, their peers in countries such as the United States or elsewhere in Europe are facing the same challenges. Even when EI professionals think they are fully implementing an FCA, their perceptions are often incorrect (Dunst et al., 2014). Home visiting programs in the United States also strongly recommend the active engagement of parents with their children during home visits (Roggman et al., 2008). Unfortunately, however, this is the case in fewer than 50% of home visits (Peterson et al., 2018).

Interestingly, in this study the professionals agreed that they would continue to use tools that allow them to promote the interaction of parents and children at home (item 11). In addition, it seems that the pandemic situation made them rethink the way they work and collaborate with families (item 12) and they realized that they needed more training in order to continue to work with families applying this more ecological approach (item 13). This is an important point, because the adoption of FCA requires the mastery of new skills and lack of training is one of the main barriers to a change of paradigm (Tomasello et al., 2010; McWilliam and García, 2016). In Spain, for some time now there have been calls for more training (Tamarit, 2015; Pereira and Oliveira, 2019; Vilaseca et al., 2019a).

As regards sociodemographic factors, the results showed that total scores on the professionals’ questionnaire could be predicted by age. Older professionals had lower total scores on the questionnaire, although the effect size can be considered as low. These findings are consistent with a study carried in Finland by Heiskanen et al. (2021) of rehabilitation professionals during the COVID-19 pandemic, in which those with the longest work experience were found to be the least likely to use telerehabilitation after the pandemic. However, our results could also be attributed to the context of the implementation of the FCA model in Spain, already discussed in the introduction section. FCP were introduced only recently and are applied inconsistently among early intervention professionals and teams. Older professionals continue to prefer child-focused models; so FCA training is a necessity if we want to achieve a change of perspective among all EI professionals.

The comparison of families’ and professionals’ perceptions of care during the pandemic present a certain amount of agreement but statistically significant differences were found in two items (items 1 and 2), on which professionals had higher average scores. As we have mentioned, parents did not clearly agree or disagree with the content of those two items, while professionals expressed full agreement. One of the issues that has important consequences for early intervention practices is the professionals’ vision of how to work with families. One of the key principles of the FCA is collaboration between parents and professionals (Dunst et al., 2000; Carlhed et al., 2003; Turnbull et al., 2004) and an insistence that parents should not be mere recipients of information, but also providers; they should participate actively and their role should not be limited to following instructions. In many European countries, including Spain, it is a priority for professionals to include parents in their intervention programs, to train them to make their own decisions and to take their perspectives much more into account (European Agency for Development in Special Needs Education, 2010). Our results are consistent with a study carried out in Spain before the pandemic with over 180 professionals and 500 families, in which professionals scored higher than families on most FCA dimensions (Escorcia-Mora et al., 2018). According to Escorcia-Mora et al. (2018), these results can be attributed to an overvaluation by professionals of their own practices and the intrinsic need to project a positive image of their work, and to a lack of training that prevents them from reflecting on other ways of intervening with families (in accordance with previous studies carried out in Spain: García-Sánchez et al., 2014; Mas et al., 2019; Vilaseca et al., 2019a). It is evident that these perceptions may vary depending on the professional’s specialization. Interestingly, several studies carried out during the pandemic found the use of telerehabilitation varied according to whether the professional was a speech and language therapist (Kraljević et al., 2020), an occupational therapist (Dahl-Popolizio et al., 2020) or another specialization.

## Conclusion and Final Remarks

The aim of this study was to assess the work that EI professionals carried out with families and children seen in EIS in Spain during the COVID-19 pandemic, and to establish whether this situation might promote a change in their practices. More specifically, we compared families’ and professionals’ perceptions of the intervention methodology used and explored the relation between these perceptions and certain sociodemographic variables.

Our main findings were that, contrary to expectations, it is not clear that the online intervention carried out during the pandemic presented significant changes in terms of the incorporation of FCP. Professionals considered that the intervention followed the defining trends of FCP, but the impression of the families was less clear-cut; although they perceived some changes with regard to the use of FCP, they noted that the intervention maintained many of the characteristics of the traditional child-centered model. The families were satisfied with the care received during the pandemic. but overall, the study shows that the professionals were not perceived as applying the standards of FCP. For professionals, the pandemic situation has highlighted the importance of the family and the involvement of all its members, and the need to promote positive parenting at home to optimize the child’s development. Although this new awareness is clearly positive, more training is still needed and policy makers in Spain should focus on ways of promoting effective change that can be extended to all EIS.

Some interesting findings were also obtained regarding the role of sociodemographic variables in the perception of the intervention model. Mothers with previous use of computer resources and who dedicated themselves entirely to caring for their children and housework were more satisfied with the intervention and observed a more widespread adoption of FCP. On the other hand, younger EI professionals perceived the online intervention as being more in line with FCP. This may indicate that, even though the objective of extending and generalizing FCP is far from being established, a change is taking place in the attitudes toward EI among younger professionals in Spain, probably due to training and to a lower adherence to more traditional models.

This study has several limitations. First, our aim of comparing the perceptions of families and professionals in relation to the intervention model during the pandemic was hindered by the fact that only six of the questions were the same for both groups because we adjusted the formulation of the items to the previous knowledge and to the characteristics of each group. The discrepancy between the items is a drawback and is an issue that needs attention in future work.

Another limitation is the sampling procedure and the sample size. Perhaps the families and the professionals who agreed to participate were particularly interested or concerned about the pandemic or had already generated discussions on the items in their professional teams. We would have liked to have been able to reach more professionals and families, but potential participants received numerous online questionnaires during the pandemic and many may have been reluctant to respond. All in all, the results are not representative of all EIS in Spain, because we know that many of them have started the transformation toward new, more systemic and ecological intervention models. This study should now be replicated with a larger number of families and professionals with a representative sample of all the regions of Spain.

Third, the study was based on self-reports and perceptions; there was no direct observation of EI professional practices. Therefore, the results should be interpreted with caution. Finally, the study’s cross-sectional design means that we cannot establish causality. We also need to qualify the term *predictor*, as used in the regression analysis. In this context, to *predict* means just to estimate total questionnaire scores based on the predictor variable scores (such as employment status, use of telematic tools, or professionals’ age), and does not necessarily imply direct causality.

In spite of these limitations, our study has several strengths. First, although the possibilities for comparison are limited, we have provided relevant data on a new topic: families’ and professionals’ perceptions of the early intervention services received and provided during the COVID-19 pandemic lockdown. The questionnaires used in this study showed both a unidimensional structure and a high internal consistency, which allows us to use them in future studies of the topic. Another strength, undoubtedly, is the focus on the impact of a critical event in spite of the obstacles that it created; the sample size is small, but it is very difficult to engage families and professionals in times of crisis. Our results show that the obligation to use the internet for the intervention led professionals to rethink some of their previous practices, raised their awareness of the interest and value of adjusting to the families’ needs, strengths, resources and aims, and increased the participation of the families inside a less directive and a more collaborative model – all of them characteristics of FCP. Although our results do not indicate a clear shift toward the use of FCP at EI services, they do suggest that the professionals’ greater focus on the family context because of the lockdown caused them to question some of their preconceptions. In this way, then, our study may help to increase the spread of FCP.

In addition, our study has implications for future early intervention programs with families. Telematic intervention during the pandemic was positively valued by parents, and managed to bring the intervention closer to the family context. Professionals saw telematic intervention as an opportunity to move toward intervention models that encourage families’ participation, their involvement in decision-making, and the deployment of strategies focused on daily routines. Professionals feel that they have made progress in this direction during the pandemic. However, as mentioned above, families have not perceived such significant changes. Several consequences follow from this. First, the use of telematic interventions does not in itself guarantee a change in the intervention model. Second, we must continue making efforts to approximate the families’ needs and professional visions, which do not always coincide. The application of innovative and remote rehabilitation interventions during the pandemic may have interesting repercussions in the post-COVID-19 scenario. Their use in daily clinical practice and in the treatment of children with neurodisabilities in their everyday environment has real potential, as long as they are family-centered and take into account the needs of the child and those of their caregivers. The use of telerehabilitation can facilitate the use of best practices, focusing on empowering families to promote the development and learning of their children with disabilities.

Research in Spain and in other countries should now continue with case studies including observation of parenting in a natural context and the provision of coaching, monitoring and feedback during in-service and online sessions. This should help to broaden our understanding of the strengths and weaknesses of online intervention in family-centered parenting practices. The benefits and limits of telerehabilitation should continue to be explored, in order to make decisions regarding its use either as a primary via of intervention or as a complementary one.

## Data Availability Statement

The original contributions presented in the study are included in the article/Supplementary Material, further inquiries can be directed to the corresponding author/s.

## Ethics Statement

The studies involving human participants were reviewed and approved by University of Barcelona’s Bioethics Commission. Written informed consent to participate in this study was provided by the participants’ legal guardian/next of kin.

## Author Contributions

RV, FF, MR, and RB made substantial contributions to conception and design, acquisition of data, analysis and interpretation of data, participated in drafting the article or revising it critically for important intellectual content, and gave final approval of the version to be submitted. All authors contributed to the article and approved the submitted version.

## Conflict of Interest

The authors declare that the research was conducted in the absence of any commercial or financial relationships that could be construed as a potential conflict of interest.

## Publisher’s Note

All claims expressed in this article are solely those of the authors and do not necessarily represent those of their affiliated organizations, or those of the publisher, the editors and the reviewers. Any product that may be evaluated in this article, or claim that may be made by its manufacturer, is not guaranteed or endorsed by the publisher.
